# Pilot trial of iBDecide: Evaluating an online tool to facilitate shared decision making for adolescents and young adults with ulcerative colitis

**DOI:** 10.1111/hex.13618

**Published:** 2022-09-26

**Authors:** Kelly A. Matula, Philip Minar, Nancy M. Daraiseh, Li Lin, Marlee Recker, Ellen A. Lipstein

**Affiliations:** ^1^ James M. Anderson Center for Health Systems Excellence Cincinnati Children's Hospital Medical Center Cincinnati Ohio USA; ^2^ Division of Gastroenterology, Hepatology and Nutrition Cincinnati Children's Hospital Medical Center Cincinnati Ohio USA; ^3^ Department of Pediatrics University of Cincinnati College of Medicine Cincinnati Ohio USA; ^4^ Division of Research in Patient Services Cincinnati Children's Hospital Medical Center Cincinnati Ohio USA; ^5^ Division of Social Services Cincinnati Children's Hospital Medical Center Cincinnati Ohio USA

**Keywords:** adolescents, decision aids, decision support tools, paediatrics, shared decision making, ulcerative colitis

## Abstract

**Introduction:**

This pilot, randomized controlled trial aimed to evaluate the usability, among adolescents and young adults (AYAs) with ulcerative colitis (UC), of a web‐based tool (‘iBDecide’) designed to facilitate shared decision making (SDM).

**Methods:**

AYAs with UC (*n* = 35) were randomized to intervention (iBDecide, *n* = 14) and control (*n* = 12) arms before a scheduled clinic visit. We measured the usability of iBDecide, SDM, preferred decision‐making style, decision conflict and intervention use.

**Results:**

Participants in the intervention group found iBDecide easy to use and agreed that it made them feel ready to participate in decision making and that they would use it to prepare for appointments. There were 130 visits to iBDecide, lasting on average 3 min, 41 s. The medication and nutrition trackers were among the most‐viewed pages. Pages specifically designed to facilitate SDM were viewed only four times. Across groups, too few participants reported making decisions during clinic visits for decision‐related measures to be reported.

**Conclusions:**

This pilot trial provides evidence for the usability of iBDecide and guidance for developing a larger‐scale trial of a combined web‐based and in‐clinic SDM intervention. Overall, iBDecide shows promise in engaging AYAs with UC in SDM and condition management.

**Patient or Public Contribution:**

Patients, specifically AYAs with UC, and healthcare providers were involved in the design of this study's intervention, iBDecide. Additionally, the research team, from study conception to manuscript writing, included a young adult with inflammatory bowel disease.

**Clinical Trial Registration**: This study was registered at clinicaltrials.gov (NCT04207008).

## 1 INTRODUCTION

Adolescents and young adults (AYA) with chronic conditions are faced with the challenge of learning to manage, and in some cases adapt to, a chronic condition while in a developmentally challenging phase of life. This is particularly true of paediatric inflammatory bowel disease (IBD) and its subtype ulcerative colitis (UC). Paediatric IBD often presents in late adolescence and 20% of all UC patients present before age 20.^1^ One of the many challenges in this and other chronic conditions is learning to engage in treatment decision making along with parents and healthcare providers, ideally via shared decision making (SDM). Experience with SDM is especially valuable in preparation for the transition to adult care providers.^2^, ^3^ For AYAs with UC, treatment decisions are complicated by tradeoffs between the positives (e.g., minimizing risks carried by active disease) and negatives (e.g., side effects, limitations to diet) associated with treatment. Further complicating matters, AYAs participating in making these decisions often lack experience with medical decision making, while simultaneously being in a developmental stage focused on increased independence from family who may have such experience.^4^, ^5^ Prior research suggests that adolescents with UC want to be involved in treatment decisions along with their parents and clinicians,^1^, ^6^ but are often not as involved as they would like to be.^6^, ^7^

A decision support tool could help address these complex issues. Decision support tools are designed to provide patients with evidence‐based information about available treatment options for a medical condition, as well as the opportunity to consider their values in choosing an option.^8^, ^9^ These tools have been shown to benefit both adult patients and parents of paediatric patients, by improving knowledge about the benefits and risks of treatment options.^8^, ^9^, ^10^ However, there are few decision tools for paediatric chronic diseases, let alone for AYA populations, and none for AYAs with UC.^11^, ^12^, ^13^, ^14^

Our team, in collaboration with a team of designers (including graphic and industrial designers and design students), previously developed a decision support tool, iBDecide,^15^ designed to meet the needs and preferences of AYAs with UC. This tool was designed with input from focus groups of AYA patients with UC^16^ and included patient and provider input in the design process. Based on the AYA input, the resulting tool differs in several important ways from standard decision support tools.^8^, ^9^, ^10^ Specifically, iBDecide is web‐based, rather than being on paper, is specifically for AYA use rather than intended for use by both young people and parents,^11^ and includes disease management functions, such as symptom tracking,^15^, ^16^ in addition to the informational and values‐elicitation materials of standard decision support tools.^8^, ^9^ The disease management tools were included based on recommendations of AYA patients.^16^ Our expectation was that the inclusion of these tools may promote general use of iBDecide, including the decision tool‐specific aspects. As described in detail elsewhere,^15^ iBDecide has eight sections: a patient profile section; tracking sections, for nutrition, symptoms and medications (including medication reminders); an ‘appointment guide’ section; a ‘My Notes’ section; and a ‘treatment options’ section that includes both general information about UC and available treatments as well as a novel interactive feature designed to increase users’ decision‐making involvement. This feature, the ‘Treatment Generator’, is an interactive chatbot that prompts the user with questions that may influence their treatment choices, such as treatment goal (e.g., end a flare, maintain remission) or willingness to receive injections, and generates a list of treatment options consistent with their responses, which they can then discuss with their clinician.^15^ iBDecide was designed to be used intermittently, rather than in one long session.

We conducted a pilot, randomized controlled trial to determine the usability, defined as the ease of use and acceptability of a system, of iBDecide. Ease of use affects the user's performance and satisfaction, while acceptability affects whether the product is used.^17^ We hypothesized that the tool would score high in usability ratings since it was designed based on adolescent input. We also planned to obtain point estimates of decision outcomes, in anticipation of a larger future trial.

## 2 METHODS

### 2.1 Study design and participants

This pilot study was a prospective, randomized controlled trial. Eligibility criteria were: AYA patients (ages 14–24) with UC who had an upcoming appointment at the hospital's gastroenterology clinic, had not participated in a prior usability testing study for the iBDecide tool^15^ and had access to a smartphone with internet capabilities. At the start of the trial, patients expected, based on chart review and discussion with their clinician, to make a treatment decision at the clinic visit were recruited. Due to more limited clinic visits and therefore low enrollment early in the COVID‐19 pandemic, we eliminated the ‘expected decision during clinic visit’ criterion to ensure an adequate sample for testing usability. Since the language of the tool was English, those unable to speak or read English were excluded.

### 2.2 Study recruitment, consent and randomization

Patients eligible to participate in the study were identified via a review of the hospital's medical record for patients with a UC diagnosis and a gastroenterology clinic appointment at least 2–3 weeks in the future. Recruitment took place between 17 February 2020 and 15 September 2020. Recruitment was ended at this time, due to funding constraints and ongoing recruitment challenges related to the COVID‐19 pandemic. Following collection of electronic written consent from participants (or from parents with assent from participants <18), participants were randomized via permuted block method to the control arm, which received usual care, or the intervention arm. Randomization was stratified by age (14–17 and 18–25 years). Participants received a $20 gift card for their participation.

### 2.3 Intervention

The intervention consisted of access to iBDecide. Participants randomized to the intervention arm were called 2–3 weeks before their clinic visit. During the call, the research staff facilitated registration on the iBDecide website and introduced its features to the participants. They were then instructed to use it however they liked going forward.

Participants in the control group were told that they would have access to iBDecide after the trial was complete but were not given further information about it.

### 2.4 Data collection

Participant data were collected via phone call 1 week after the clinic appointment. Additionally, iBDecide usage data were collected via Google Analytics version 4 (Google LLC) from iBDecide registration to 3 months after the participant's clinic visit. Usage data were collected for individual IP addresses and in aggregate. The last data were collected 3 months after enrollment of the last participant, 28 November 2020.

After the clinic appointment, the participant's physician was sent an electronic link to the postvisit survey. A reminder email was sent 2–3 days following the initial survey link, followed by delivery, a week later, of a hard copy to any nonresponding physicians. Physicians were blinded to participants' study arm assignment.

### 2.5 Measures

#### 2.5.1 Survey data

The primary outcome was the usability of iBDecide, measured using an 18‐item survey. This survey was developed by adapting measures evaluating other SDM interventions^18^, ^19^ and the system usability scale (Cronbach's *α* = .911; concurrent validity with user‐friendliness *r* = 0.806; reliability = 0.9; correlation with successful task completion *r* = 0.486)^20^, ^21^ and removing redundant items. All items, shown in Table 2, were scored on a 5‐point Likert‐type scale where 1 is completely disagree and 5 is completely agree. To assess decision outcomes, two additional items at the end of this scale asked participants if they had used iBDecide during the visit and, if so, for what purpose (e.g., pull‐up questions to ask the doctor).

Decision outcomes were measured for both arms of the study. All participants who reported making a decision during the index appointment were asked to complete the Control Preferences Scale^22^ and the Control Preferences Scale‐post^23^ to assess how the decision was made and how they would have liked it to be made. They also completed the 10‐item, 3‐option version of the Decision Conflict Scale (DCS),^24^, ^25^ as well as the SDM‐Q9^26^ to report the level of SDM that occurred during their clinic visit.

Demographic information including age, race, gender, UC severity and prior use of health apps was collected from all participants via a self‐report survey.

Physicians reported whether a treatment decision was made during the participant's visit; if yes, they reported the amount of SDM that occurred using the physician version of the SDMQ‐9 scale (SDMQ‐9‐doc).^26^ They also reported whether the patient had used iBDecide (Yes, No or Unsure). Clinicians who indicated that their patients had used iBDecide were given an adapted version of the patient usability scale to complete.

#### 2.5.2 iBDecide usage data

Individualized usage data could only be collected by IP address, not by the participant; the same participant accessing iBDecide on multiple devices (e.g., a phone and a tablet) would have an IP address for each device. For each IP address, the number of sessions and average session duration were collected.

Aggregate usage data were available across the site as a whole and across specific pages within the site. These data include the number and durations of sessions, the pages viewed during each session, and the time spent on each page. For specific pages, we can see both the number of times it was viewed and the number of unique sessions during which it was viewed (e.g., excluding multiple visits to the page by a participant within the same website visit).

### 2.6 Sample size

As this was a pilot study, the sample size was based on prior usability studies^27^, ^28^ and guidelines for pilot studies,^29^ not a power calculation. To prepare for a future study with 80% power to detect a small effect size (0.1 ≤ Cohen's *d*  <  0.3) with two‐sided 5% significance, this pilot study was planned for 40 participants with 20 per arm.^30^

### 2.7 Analysis

Participant demographics were summarized using median and interquartile ranges for continuous variables and frequencies and percentages for categorical variables. Depending on the variable types, two‐sample *t*‐test, Wilcoxon rank‐sum test, *χ* ^2^ test or Fisher‐Freeman‐Halton exact test were performed, as appropriate, to compare each demographic variable between the intervention group and control group. Unless otherwise noted, all tests were conducted at the unadjusted *α* = .05 level.

The usability questions were analysed using descriptive statistics. Given the small samples, nonparametric two‐sample tests were chosen for the outcome measures to be completed by both participant groups. Group differences in the DCS and SDMQ‐9 were analysed using Wilcoxon rank‐sum tests. Decision preference congruence (comparing CPS to CPS‐post) was analysed using Fisher's exact test. For the DCS and SDMQ‐9 (both patient and doctor), *t*‐tests were used to study the differences in the total scores between the treatment and control groups. Potential demographic effects on these scores would be examined using Pearson correlation, *t*‐test, or mixed‐effects analysis of variance, as appropriate. All data were analysed employing SAS statistical software, version 9.4 (SAS Institute).

Counts for views of different pages of iBDecide as well as per‐page and per‐client averages were determined.

## 3 RESULTS

### 3.1 Participants and demographics

**Figure 1:**
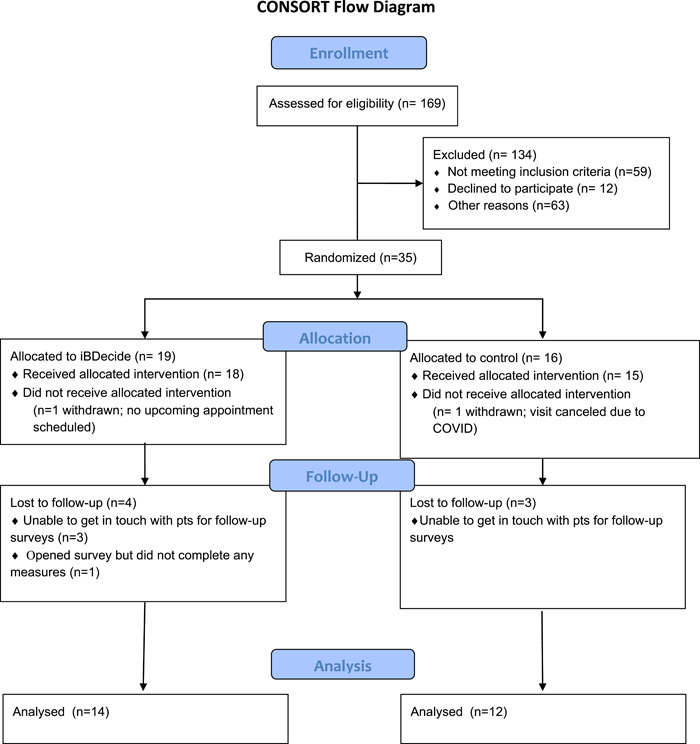
CONSORT diagram

A total of 35 participants were initially enrolled, with 16 in the control arm and 19 in the intervention arm (see Figure 1). Both the intervention and control groups had one participant withdraw before receiving their allocated intervention and three participants lost to follow‐up; an additional participant in the intervention group did not complete any follow‐up measures and was treated as having withdrawn after the start of the intervention. Demographic data and other details of the 26 participants with analyzable data are shown in Table 1. Overall, 15 (58%) participants were female and 96% were Caucasian. Twenty‐five (96%) patients reported mild or moderate UC and 19 (73%) had used some medications or treatments for UC. No significant differences were found in patient demographics between the intervention and control arms.

**Table 1:** Participant characteristics (*N* = 26)

	iBDecide (*n* = 14)	Control (*n* = 12)	*p* Value
Age in years, median (IQR)	18.9 (17.2–20.6)	17.6 (16.8–20.9)	0.65
Age category, *n* (%)			0.43
14–17 years	6 (43)	7 (58)	
18–25 years	8 (57)	5 (42)	
Gender, *n* (%)			0.46
Male	5 (36)	6 (50)	
Female	9 (64)	6 (50)	
Race, *n* (%)			0.46
White	14 (100)	11 (92)	
Black/African American	0 (0)	1 (8)	
Other^a^	2 (14)	0 (0)	
Hispanic, *n* (%)			1.00
Yes	0 (0)	0 (0)	
No	14 (100)	12 (100)	
Self‐reported disease severity, *n* (%)			0.15
Mild	3 (21)	6 (50)	
Moderate	10 (71)	6 (50)	
Severe	1 (7)	0 (0)	
Medications/treatments for UC, *n* (%)			0.77
Biologics	3 (21)	5 (42)	
Aminosalicylates	3 (21)	2 (17)	
Small molecule	1 (7)	0 (0)	
Immunomodulators	0 (0)	0 (0)	
Surgery	0 (0)	0 (0)	
Herbal or natural remedies	0 (0)	0 (0)	
Other	3 (21)	2 (17)	
None	0 (0)	0 (0)	
Have used other apps for UC, *n* (%)			0.65
Yes	4 (29)	2 (17)	
No	10 (71)	10 (83)	

Abbreviations: IQR, interquartile range; UC, ulcerative colitis.

^a^ Two patients checked both ‘White’ and ‘Other’.

### 3.2 Primary outcome measures

#### 3.2.1 iBDecide usability

As shown in Table 2, 13 of 14 intervention group participants provided usability data. All items related to positive aspects of iBDecide had median scores above 3, indicating that participants overall found iBDecide easy to use and beneficial. Items focused on its ease of use, the helpfulness of the information and how quick it would be to learn all had median scores of 5. In fact, no items making positive statements about iBDecide had median scores below 4, while those making negative statements about it all had median responses of 1 or 2.

**Table 2:** iBDecide usability ratings

Usability measures^a^	Median (IQR)
1. iBDecide has the right amount of information about UC treatment choices.	4 (4–4)
2. The information about UC treatment in iBDecide is clear.	4 (4–5)
3. The information about UC treatment choices in iBDecide is helpful.	5 (4–5)
4. I would like to get information about other medical treatment choices in the same way that I got information about UC treatment choices from iBDecide.	4 (4–4)
5. Using iBDecide made me feel more ready to participate in decisions related to treating my UC.	4 (3–5)
6. Using iBDecide made me feel more prepared for my doctor's appointment.	4 (4–5)
7. iBDecide is too complicated.	2 (1–2)
8. I thought IBDecide was easy to use.	5 (4–5)
9. I think I would need the support of a technical person to use the iBDecide app.^b^	1 (1–2)
10. I thought the information in iBDecide was not consistent.^b^	2 (1–2)
11. I would imagine that most people would learn to use iBDecide very quickly.	5 (4–5)
12. I found iBDecide challenging to use.^b^	2 (1–2)
13. The different parts of iBDecide worked well together.	4 (4–4)
14. I felt very confident using iBDecide.	4 (4–5)
15. I needed to learn a lot of things before I could start using iBDecide.^b^	2 (2–2)
16. I would use iBDecide as part of my daily routine.	4 (3–4)
17. I would use iBDecide to prepare for a doctor appointment.	4 (4–5)
18. I would recommend iBDecide to other patients with UC.	4 (4–5)
21. Did you use iBDecide during your clinic visit? (*n*)	
Yes	2 (15%)
No	11 (85%)
22. If yes, which of the following did you do during the visit (check all that apply)? (*n*)	
Look at information about treatment options	0
Pull up questions to ask my doctor	1
Tell or show my doctor information I had been tracking about symptoms	2
Tell or show my doctor information I had been tracking about food	1
Record my medications	0
Record my next clinic visit	0
Other (specify)	0

Abbreviations: IQR, interquartile range; UC, ulcerative colitis.

^a^ Unless otherwise noted, responses range from 1 (completely disagree) to 5 (completely agree).

^b^ Reverse scored.

Two participants indicated that they used iBDecide during their clinic visit. Both of these said they used it when telling their physician symptom information they had been tracking before the clinic visit. Additionally, one of these two participants reported using it to pull up questions to ask their physician, while the other participant accessed food tracking information.

Physicians returned survey data for 14 intervention participants and 9 control participants. No physicians reported knowing that their patient had used iBDecide. Therefore, we have no physician acceptability data.

#### 3.2.2 iBDecide usage data

General usage data for iBDecide are shown in Table 2. Among the 14 participants in the intervention group, there were 23 IP addresses that used iBDecide at least once after initially logging in under the instruction of the research staff. We do not have data on how these IP addresses map across participants or different locations or devices, so we cannot say how many used it on multiple devices or in multiple locations.

The number of times a given IP address (participant on a given device) viewed the app ranged from 2 to 29 times (median = 4). In total there were 130 visits to iBDecide. An average of 6.8 pages were viewed per session, and the average session duration was 3 min 41s. Of the 112 sessions lasting longer than 30 s, nearly half were over 3 min.

The three most viewed sections, after the log‐in page, were the main tracking interface and the specific pages to track medications and nutrition. Both the Treatment Generator and Compare Medications pages were viewed during only four sessions.

### 3.3 Secondary outcome measures—Decision‐making data

Only 10 participants (4 in the iBDecide arm and 6 in the control arm) reported a decision was made during their appointment, and only 7 clinician surveys indicated that a decision was made during the visit. With so few decisions made during clinic visits, no decision‐making data are reported.

## 4 DISCUSSION AND CONCLUSION

### 4.1 Discussion

The goal of our study, consistent with recommendations for pilot trials,^29^ was to test the usability of iBDecide and pilot protocols for a future larger‐scale trial. Overall, usability, including acceptability, and ratings for iBDecide were high. Our data demonstrated that participants who used the tool felt better prepared for their doctor's appointments and to make potential treatment decisions about their conditions. They also reported they would continue to use iBDecide to help prepare for future appointments and would recommend it to others. While the anticipated daily use of iBDecide was lower than agreement with these other statements, overall user responses indicated iBDecide met the needs for which it was designed.

These results are notable given the small number of decision support tools available either for UC or targeted to AYA populations. In terms of UC decision aids, a tool focused on surgery for UC exists,^31^ but no usability or acceptability data for it are available. A more general UC decision aid is in development but is intended for adults, and limited validation data are available.^32^ More generally, there is a lack of AYA‐targeted decision‐support tools. A recent review found only eight paediatric decision support tools that had been tested with adolescents.^11^ Only one specifically targeted young adults (i.e., did not cap enrollment at 18 nor include concurrent use by parents); it focused on depression, and usability and acceptability data are not available.^33^, ^34^ A few other recent studies of decision aids for paediatric chronic conditions do exist,^12^, ^35^ as well as one for AYA cancer patients focused on fertility‐preservation treatments,^13^, ^14^ but only one provides usability data.^13^ In comparison to other web‐based decision support tools, iBDecide has similar usability and acceptability.^36^, ^37^, ^38^, ^39^, ^40^ This similar usability corroborates not only iBDecide's standard decision support functions but also its novel condition management features.

The limited use of aspects of iBDecide designed to assist with treatment planning and decision making may be due to participants recognizing that they were not likely to make a decision at their upcoming appointment, as evidenced by limited reports of decision making at clinic visits. This low utilization may also be due to uncertainty about how best to use iBDecide in conjunction with clinic appointments, particularly how their doctor would react to their using iBDecide as this was the first clinic visit at which they had it available. Additionally, given the dynamic nature of UC,^41^ many medical decisions are made outside of clinic appointments, such as after an endoscopy procedure or on the phone. These diverse opportunities for decision making should be considered for future implementation of iBDecide. It is also possible that participants were simply more interested in the tracking portions of iBDecide. In fact, AYA participants in the iBDecide development focus groups were more interested in including tracking tools than decision‐making tools,^15^ despite our preliminary work^16^ indicating AYA desire for an AYA‐targeted decision tool.

A limitation of this study is that the components most explicitly designed to facilitate decision‐making involvement—the Treatment Generator and Compare Medications tools—were rarely used. To more fully evaluate these features, AYA attitudes toward them, and their effects on decision involvement, further research with iBDecide should focus on patients expected to make a decision at upcoming appointments (e.g., those recently diagnosed or with increased symptoms). Additionally, since few participants used iBDecide during their clinic visit and no clinicians indicated awareness of such use, we were not able to gather physician acceptability data or see how iBDecide fits into clinic visits. Also, we did not reach our recruitment goal (likely due to the COVID‐19 pandemic). However, with 26 participants, this pilot study can prepare a study detecting medium effect size.^30^ The 2–3 weeks study window before participants' clinic appointments may have been too short of a time for them to integrate an app into their daily lives and we were unable to track individual participants across different devices. Future testing of iBDecide should integrate more advanced methods for tracking site usage to enable tracking by user, and provide more time for the participants to use the app. Finally, because the usability questions were assessed via telephone, there may have been social desirability effects.

The next step in our evaluation of iBDecide, a multisite, factorial design trial, will address these limitations and test iBDecide alone, and in combination with decision conversation cards, our team developed for use by patients and physicians during clinic visits.^42^ These cards include the same information as the iBDecide Treatment Generator. The fact that participants in the current study used iBDecide but did not discuss it with their physicians suggests that combining the online tool with a conversation tool, like the cards, would be beneficial. Such a combined intervention would include both decision preparation and conversation support in a synergistic approach to better facilitate AYA engagement in medical decision making.

Our results also suggest potential improvements that could be made to iBDecide. For instance, since many participants indicated that iBDecide was helpful in preparing them for their appointment, it may be useful to add functionality allowing iBDecide to prompt patients to use it before an appointment. Likewise, integration with the electronic medical record could facilitate use during the clinical encounter. For example, patient‐provided nutrition or tracking data could be imported into the health record, and the physician could be prompted to discuss these with the patient during the visit.

### 4.2 CONCLUSION

Overall, the results of this pilot study suggest that AYA patients with UC found a web‐based decision support tool targeted toward them informative and easy to use. This study provided usability information that will inform the planning of a larger trial to investigate the impact of a combined shared decision‐making intervention (web‐based tool plus decision facilitation cards) on the treatment involvement of AYA with UC. Such involvement is essential for them as they transition into adult care. The iBDecide intervention shows promise in involving AYA with UC in their disease management and related decisions. Before clinical implementation, it is important iBDecide is evaluated in a larger, multisite trial. If found to be effective at facilitating SDM, it could easily be adapted for other chronic conditions.

## AUTHOR CONTRIBUTIONS

**Kelly A. Matula**: Writing–original draft preparation. **Philip Minar**: Writing–review and editing; methodology. **Nancy M. Daraiseh**: Writing–review and editing; methodology. **Li Lin**: Writing–review and editing; data curation; formal analysis. **Marlee Recker**: Writing–review and editing. **Ellen A. Lipstein**: Conceptualization; supervision; methodology; writing–review and editing.

## CONFLICT OF INTERESTS

The authors declare that there are no conflict of interests.

## ETHICS STATEMENT

This study was approved by the institutional review board of Cincinnati Children's Hospital Medical Center (Approval number 2018‐4800).

## Data Availability

The data that support the findings of this study are available on request from the corresponding author. The data are not publicly available due to privacy and IRB restrictions.
